# Роль менопаузальной гормональной терапии в инициации плейотропного (антивозрастного) эффекта посредством замедления репликативного клеточного старения у женщин (обзор литературы)

**DOI:** 10.14341/probl12895

**Published:** 2022-03-23

**Authors:** О. Р. Григорян, Т. М. Фролова, Р. К. Михеев, Е. В. Шереметьева, Ю. С. Абсатарова, Ж. А. Ужегова, Е. Н. Андреева, Н. Г. Мокрышева

**Affiliations:** Национальный медицинский исследовательский центр эндокринологии; Национальный медицинский исследовательский центр эндокринологии; Национальный медицинский исследовательский центр эндокринологии; Национальный медицинский исследовательский центр эндокринологии; Национальный медицинский исследовательский центр эндокринологии; Национальный медицинский исследовательский центр эндокринологии; Национальный медицинский исследовательский центр эндокринологии; Московский государственный медико-стоматологический университет им. А.И. Евдокимова; Национальный медицинский исследовательский центр эндокринологии

**Keywords:** менопауза, теломеры, менопаузальная гормональная терапия, эпигенетика

## Abstract

На современном этапе гендерные различия доказывают, что процесс демографического старения населения более характерен для лиц женского пола. Менопауза, или климактерий, — это преддверие старости, но не сама старость, а закономерный физиологический процесс, который невозможно остановить, однако необходимо помочь женщине чувствовать себя уверенно и комфортно в любой ситуации. Менопаузальная гормональная терапия (МГТ) за счет эстрогенового компонента оказывает противовоспалительное, антиоксидантное действия и способствует экспрессии теломеразы, что в совокупности изменяет гомеостаз и целостность теломер. Применение МГТ в течение 5 лет и более способно не только значительно изменить качество жизни, но и увеличить ее продолжительность. Поиск литературы проводили в отечественных (eLibrary, CyberLeninka.ru) и международных (PubMed, Cochrane Library) базах данных на русском и английском языках. Приоритетным являлся свободный доступ к полному тексту статей. Выбор источников был приоритетен периодом с 2019 по 2021 гг. Однако с учетом недостаточной изученности выбранной темы выбор источников датировался с 1989 г.

## Abstract (en)

Present worldwide healthcare researches prove that female patients are more sensitive to the population aging. Menopause or climacteria (climax) — is not as ageing itself, but a physiological unstoppable process. The main task for a physician is to improve life quality for female despite of ageing problems. Menopausal hormone therapy (MHT) due to the estrogen component has an anti-inflammatory, antioxidant effect and promotes the expression of telomerase, which together changes the homeostasis and integrity of telomeres. The use of MHT for five years or more can not only significantly change the quality of life, but also increase its duration. Literature search was carried out in national (eLibrary, CyberLeninka.ru) and international (PubMed, Cochrane Library) databases in Russian and English. The priority was free access to the full text of articles. The choice of sources was prioritized for the period from 2019 to 2021. However, taking into account the insufficient knowledge of the chosen topic, the choice of sources dates back to 1989.

## ВВЕДЕНИЕ

За последнее столетие средняя продолжительность жизни человека увеличилась на фоне постепенного падения темпов рождаемости. Демографическое старение населения наблюдается не только в РФ, но и в большинстве стран мира. По прогнозу ООН, к 2050 г. доля людей старше 65 лет может удвоиться, а к 2100 г. — утроиться и достигнуть почти 25% населения мира [1]. Однако жить дольше — не равно жить здоровее [2].

Само по себе старение — не болезнь. На этот процесс мы повлиять не можем, более того, ряд эпигенетических факторов его ускоряет, поэтому профилактикой старения следует заниматься с рождения. В настоящий момент Индустрия здорового долголетия активно развивается, разрабатывая различные стратегии.

В России, по статистическим данным, около 40% граждан пенсионного возраста продолжают работать, а 50% из них — женщины в постменопаузальном периоде. «Менопауза, или климактерий, — это преддверие старости, но не сама старость, а закономерный физиологический процесс, который невозможно остановить, однако необходимо помочь женщине чувствовать себя уверенно и комфортно в любой ситуации» [3].

Существует несколько теорий старения организма — одна из них, наиболее актуальная, теломеразная [4].

## ТЕЛОМЕРЫ, ТЕЛОМЕРАЗНАЯ АКТИВНОСТЬ

Теломеры — это специализированные концевые участки линейных хромосом эукариот. ДНК теломер представлена повторяющимися нуклеотидными последовательностями, богатыми гуанином — TTAGGG [5]. Именно с этими тандемными повторами связываются белки, образуя нуклеопротеидный комплекс. При рождении длина теломерной ДНК соматических клеток, таких как лимфоциты, составляет от 8 до 14 килобаз (тысяч пар оснований) [6]. При каждом делении клетки удаляется от 50 до 100 пар оснований этой теломерной ДНК в связи с неполной репликацией 3’-концов [7]. При достижении критического количества пар нуклеотидов (для лимфоцита — 4 килобазы) клетка подвергается апоптозу. Однако теломеры — это не только «биологические часы» конкретной клетки, но и индикатор состояния организма в целом.

Процессу неизбежного укорочения теломер при каждом делении клетки противодействует особый фермент — теломераза. А в некоторых бессмертных клетках присутствует альтернативный механизм удлинения теломер [8]. Теломераза состоит из двух главных субъединиц: теломеразной обратной транскриптазы (ТОТ) и теломеразной РНК (тРНК). Взаимодействуя, эти два компонента выполняют классическую функцию теломеразы — элонгацию теломер [9]. Первоначально считалось, что теломераза присутствует лишь в небольшом количестве клеток: зародыша, плода, а также в клетках опухоли. Однако было показано, что субъединица теломеразы ТОТ также присутствует и в ряде тканей (клеток) с низким репликативным потенциалом, где она противодействует апоптозу и поддерживает клеточный окислительно-восстановительный гомеостаз [10]. ТОТ контролирует уровень активных форм кислорода (АФК) в цитозоле и в митохондриях, защищает митохондриальную ДНК (мДНК) и поддерживает концентрацию антиоксидантных ферментов. Снижение количества митохондриальной ТОТ может вносить свой вклад в патогенез развития ряда заболеваний, ассоциированных с окислительным стрессом. Повышение уровня митохондриальной ТОТ может иметь терапевтическое значение.

Известно, что ряд факторов транскрипции играет роль в регуляции промотора ТОТ, среди них выделяют и рецептор эстрогена. Таким образом, эстрогены, связываясь со своим рецептором, могут регулировать уровень ТОТ внутри клетки.

Помимо механизма неизбежного укорочения, выделяют также механизмы ускоренного укорочения теломер. К ним относятся как генетические, так и эпигенетические факторы: метилирование ДНК, модификации гистонов, РНК-интерференция [11]. Наиболее частой причиной развития эпигенетических изменений, приводящих в итоге к укорочению длины теломер (ДТЛ), считается оксидативный стресс (ОС) [12]. Было отмечено, что при ряде заболеваний, ассоциированных с ОС, наблюдается укорочение теломер [13]. По данным некоторых авторов, короткая ДТЛ ассоциирована с более выраженными нарушениями углеводного обмена и степенью инсулинорезистентности [14].

ОС возникает в результате дисбаланса между производством АФК и клеточной антиоксидантной защитой и вносит свой вклад в патогенез различных заболеваний, таких как атеросклероз, сахарный диабет, ожирение, артрит, болезнь Альцгеймера и др. [15][16]. В большинстве случаев основными источниками АФК являются воспаление и/или митохондриальная дисфункция.

До сих пор неясно, почему именно теломеры в большей степени по сравнению с остальной ДНК повреждаются под воздействием АФК: то ли вследствие повышенной восприимчивости, то ли из-за недостаточной репарации. Выделяют несколько возможных механизмов, лежащих в основе повышенной чувствительности концевых участков хромосом к АФК. 1) Из всех азотистых оснований гуанин наиболее восприимчив к окислению, а теломеры в значительной степени обогащены именно им. При окислении гуанина образуется 8-оксогуанин — один из основных биомаркеров повреждения ДНК. 2) Нуклеотидные последовательности TTAGGG являются предпочтительными сайтами для связывания ионов железа. Как известно, при их взаимодействии с пероксидом водорода образуются гидроксильные радикалы (реакция Фентона), приводящие к однонитевым разрывам ДНК теломер. Так, в нескольких исследованиях сообщалось, что после воздействия на клетки ОС в теломерах наблюдалось большее количество 8-оксогуанина и однонитевых разрывов ДНК по сравнению с микросателлитными повторами и основной массой геномной ДНК. 3) Теломеры обогащены особым антиоксидантом, поглощающим пероксид водорода, — пероксиредоксином 1, потеря которого приводит к преимущественному повреждению теломер. Что касается недостаточного восстановления ДНК теломер от повреждения азотистых оснований, существует теория, согласно которой, один из компонентов белкового комплекса, «шелтерин» (TRF2), препятствует полноценной эксцизионной репарации оснований [17].

Главным источником АФК являются митохондрии, однако в норме их избыточному негативному воздействию на ДНК препятствуют внутренние защитные механизмы — так называемые развернутые митохондриальные белки (UPRmt), а также, согласно некоторым данным, ТОТ. Было показано, что при кратковременном окислительном стрессе ТОТ экспортируется из ядра и накапливается в митохондриях [18]. Тем не менее при перегрузке клетки питательными веществами, например, при ожирении, защитные механизмы ломаются и развивается митохондриальная дисфункция. Таким образом, данное явление — один из ключевых механизмов развития ряда хронических заболеваний, ассоциированных с хроническим воспалением и ОС.

При голодании и активной физической нагрузке отмечается тенденция к уменьшению отношения восстановленного никотинамидадениндинуклеотида к окисленному (НАД+/НАДН), что снижает потенциал генерации АФК. При повышенном поглощении питательных веществ в совокупности с сидячим образом жизни снижается потребность в АТФ, соответственно, отношение НАДH/НАД + увеличивается, что, в свою очередь, способствует увеличению образования АФК, вызывает повреждение мДНК и приводит к дисфункции митохондрий. Это, в свою очередь, вносит вклад в клеточное старение и отражается на ДТЛ ядерной ДНК [19].

Кроме того, верно и обратное: не только воспаление приводит к укорочению теломер, но и укорочение теломер приводит к развитию системного воспаления и ускоренного старения. Так, в исследовании на рыбках Данио с очень короткими теломерами наблюдалось повышенное распространение меланомы [20]. То есть, помимо клеточно-автономной роли коротких теломер в нестабильности генома, укорочение теломер с возрастом вызывает системное хроническое воспаление, ведущее к развитию онкологических заболеваний. В некоторых исследованиях представлена обратная корреляция ДТЛ, неблагоприятных сердечно-сосудистых событий и смертности от всех причин [21].

## СВЯЗЬ ПЛЕЙОТРОПНОГО ЭФФЕКТА МЕНОПАУЗАЛЬНОЙ ГОРМОНАЛЬНОЙ ТЕРАПИИ С ТЕОРИЕЙ «ТЕЛОМЕРАЗНОГО СТАРЕНИЯ»

Общеизвестный факт: женщины живут дольше, чем мужчины, и это имеет прямую корреляцию с ДТЛ. Большую ДТЛ женщин объясняют длительным воздействием высоких концентраций эстрогена вплоть до менопаузы [22]. Логичным продолжением изучения влияния эстрогена на ДТЛ стал вопрос связи длины теломер и времени наступления менопаузы. В исследование была включена выборка из 486 женщин европеоидной расы ≥65 лет, менопауза у которых произошла естественным путем. Были учтены такие социальные аспекты, как возраст, материальное положение, образование, индекс массы тела (ИМТ), физическая активность, курение и употребление алкоголя. При каждом увеличении на 1 килобазу ДТЛ средний возраст наступления естественной менопаузы увеличивался на 10,2 мес (95% доверительный интервал (ДИ) 1,3–19,0). Однако при исключении из выборки женщин с менопаузой, наступившей ранее 40 лет, ассоциация уменьшилась до 7,5 мес (95% ДИ -0,4–15,5). Таким образом, были сделаны выводы о том, что у женщин с самой большой ДТЛ менопауза произошла на 3 года позже, чем у женщин с самой короткой ДТЛ. Кроме того, математический анализ был проведен и у 179 женщин с хирургической менопаузой, однако в этой группе связи между возрастом последней менструации и ДТЛ практически не наблюдалось [23]. Данные результаты указывают на то, что ДТЛ может служить маркером наступления естественной менопаузы.

Как известно, ДТЛ — это генетически обусловленный параметр, воздействие эпигенетических факторов отражается на ней в меньшей степени. Даже у пожилого индивидуума разница в ДТЛ между клетками различных тканей значительно меньше, чем межиндивидуальная вариабельность. Таким образом, правдоподобность гипотезы исследования основывается на двух предположениях: (1) ДТЛ в клетках гранулезной оболочки яичника коррелирует с ДТЛ в яйцеклетках, сокращение которых вызывает менопаузу, и (2) постменопаузальные ДТЛ прямо пропорциональны пременопаузальным. Иными словами, ДТЛ после менопаузы в лейкоцитах, по-видимому, отражает ДТЛ в пременопаузе в яйцеклетках.

Вероятно, неслучайно критерием включения в исследование стала европеоидная раса участниц, поскольку величина и направление связи между ДТЛ и возрастом при наступлении менопаузы варьируют в зависимости от расовых и этнических групп. Так, например, у жителей США негроидной расы наблюдалась прямо противоположная корреляция: увеличение ДТЛ на 1 килобазу ассоциировалось с наступлением менопаузы на 17,3 мес раньше (95% ДИ -39,8–5,5) [23]. Таким образом, гипотеза о том, что более короткая ДТЛ является предиктором более раннего возраста наступления естественной менопаузы, была опровергнута [24].

В 2016 г. было выдвинуто предположение о связи между материнским возрастом при рождении последнего ребенка и продолжительностью жизни самих матерей. Исследователи ориентировались на ДТЛ. Выборка была представлена 387 женщинами, родившими хотя бы одного ребенка и дожившими до пятого процентиля своей когорты, умершими до того, как пятый процентиль их когорты умер. Была выявлена закономерность между возрастом при рождении последнего ребенка и ДТЛ. Так, ДТЛ женщин, последние роды которых произошли в 33 года и позже, чаще оказывалась во второй и третьей тертилях, по сравнению с ДТЛ женщин, родивших своего последнего ребенка до 29 лет. Таким образом, возраст последних родов прямо пропорционален продолжительности жизни женщины [25]. В недавнем исследовании уже на большей выборке участниц (1232) вновь была подтверждена эта закономерность [26].

В настоящее время известно о положительном воздействии эстрогена на костную систему женщин. При вступлении в перименопаузальный период резко повышается риск развития остеопороза, с последующим возникновением низкотравматических переломов.

В 2007 г. проводилось исследование взаимосвязи между ДТЛ, минеральной плотностью костной ткани и остеопорозом на когорте 2150 женщин из популяции близнецов в возрасте 18–79 лет. Была произведена корректировка на возраст, ИМТ, статус менопаузы, курение, прием менопаузальной гормональной терапии (МГТ). Исследователи получили следующие результаты: ДТЛ положительно коррелировала с минеральной плотностью костей позвоночника (p<0,005), предплечья (p<0,013), но не шейки бедренной кости (p<0,06). Более длинные теломеры были связаны с уменьшением риска клинического остеопороза на двух или более участках (отношение шансов (ОШ) 0,594; 95% ДИ 0,42–0,84; р<0,003), а у женщин старше 50 лет клинический остеопороз ассоциировался с меньшей ДТЛ, что эквивалентно 5,2 годам теломерного старения. Следовательно, короткая ДТЛ независимо связана с уменьшением минеральной плотности кости и наличием остеопороза у женщин. В связи с вышеперечисленным ДТЛ может быть маркером биологического старения кости [27]. Данное исследование косвенно указывает на взаимосвязь эстрогенов и ДТЛ, поскольку при развитии остеопороза в перименопаузальный период в организме женщины определяется низкий уровень эстрогенов.

Неоднократно авторы задавались вопросом: помогает ли приверженность здоровому образу жизни замедлить укорочение ДТЛ? Североамериканское общество менопаузы в 2012 г. изучило связь регулярных физических упражнений и ДТЛ. Вряд ли исследование можно считать достоверным, так как в качестве экспериментальной группы рассматривалась объективно малая выборка (44 женщины в постменопаузе). Измерялись масса тела, рост, артериальное давление, окружность талии и бедер. Определяли количество копий мДНК и ДТЛ. Привычные физические упражнения определялись как комбинированные упражнения, выполняемые в течение 60 минут более 3 раз в неделю более 12 мес. Средний возраст всех участников составил 58,11±6,84 года. У активно занимающихся женщин уровни триглицеридов в сыворотке крови были существенно ниже, а уровни холестерина липопротеинов высокой плотности, число копий мДНК и ДТЛ выше [28]. Роль правильного питания в поддержании ДТЛ на первый взгляд кажется неоднозначной. Тем не менее в 2020 г. был опубликован метаанализ, включивший 8 оригинальных поперечных исследований. Число участников составило 13 733 человека из 5 стран. Положительная корреляция между соблюдением средиземноморской диеты и ДТЛ наблюдалась во всех исследованиях, за исключением проведенных только среди мужчин. Настоящий метаанализ поперечных исследований показывает, что более высокая приверженность средиземноморской диете ассоциирована с большей ДТЛ [29]. Boccardi V. и соавт. получили похожие результаты на когорте пожилых людей после логистического регрессионного анализа, который включал возраст, пол, курение и тип питания. Таким образом, применение МГТ, равно как и приверженность здоровому образу жизни, вероятно, помогает замедлить укорочение ДТЛ у женщин после наступления менопаузы. В то же время для подтверждения этой связи необходимы более масштабные и высококачественные проспективные исследования и клинические испытания [30][31].

В 2018 г. Ш. Сайбан с соавт., изучая связь между ДТЛ и преждевременной недостаточностью яичников (ПНЯ), ставит под сомнение достоверность гипотезы о положительном влиянии эстрогенов на ДТЛ. В процессе исследования генома выполнили забор крови у 40 пациенток с идиопатической ПНЯ и у 40 здоровых женщин репродуктивного возраста. Относительную ДТЛ оценивали с помощью количественной ПЦР с использованием теломерных праймеров (отношение T (теломер)/S (одноколонный ген)). Результаты данного исследования показали возможную связь между удлинением теломер и ПНЯ. Авторы выделяют несколько вероятных механизмов, обуславливающих наблюдаемое явление: 1) из-за дефектов функции яичников фолликулы делятся в меньшей степени и, следовательно, меньше укорачивается ДТЛ по сравнению с группой контроля; 2) внезапное высвобождение большого количества яйцеклеток перед наступлением менопаузы/истощением яичников может привести к кратковременному повышению уровня эстрогена и, соответственно, к удлинению теломер (за счет множественных противовоспалительных и антиоксидантных свойств эстрогенов); 3) если пациентка с ПНЯ уже принимает гормональную терапию по поводу бесплодия, то это может привести к замедлению скорости деления клеток яичников и, следовательно, способствовать удлинению теломер [32].

Одно из первых исследований, изучившее взаимосвязь между воздействием эндогенного эстрогена, ДТЛ, теломеразной активностью у женщин в постменопаузе, получавших МГТ в течение как минимум 1 года или дольше, датировано 2010 г. ДТЛ измеряли с использованием количественного метода ПЦР и теломеразы по протоколу TRAP (Telomere-Repeats Amplification Protocol) в мононуклеарных клетках периферической крови (РВМС). Субъектами исследования были 53 женщины в постменопаузе (35 с физиологической и 18 с хирургической менопаузой). Длительность репродуктивных лет жизни, рассчитанная как разница между возрастом менопаузы и возрастом менархе, использовалась как показатель продолжительности воздействия эндогенного эстрогена. Длительность приема МГT была мерой, используемой для оценки продолжительности воздействия экзогенного эстрогена. Обнаружили, что более длительное воздействие эндогенного эстрогена ассоциировалось с большей ДТЛ и с меньшей активностью теломеразы. Продолжительность репродуктивного периода также была обратно пропорциональна короткой ДТЛ и высокому уровню активности теломеразы (ОШ=0,78; 95% ДИ 0,63–0,97; p=0,02), однако на основании этих данных невозможно было напрямую оценить связь эндогенных эстрогенов с активностью теломеразы, поскольку данный параметр измерялся спустя годы после наступления менопаузы. Длительность приема МГТ не коррелировала с активностью теломеразы и ДТЛ. Результаты исследования показали, что воздействие эндогенных эстрогенов может быть ассоциировано с замедлением клеточного старения [33].

В дальнейшем исследователи использовали более крупные выборки. Ученые из Южной Кореи изучали влияние долгосрочной МГТ на ДТЛ в постменопаузе. Для участия были отобраны 130 женщин в возрасте от 55 до 69 лет, в дальнейшем разделенные на две группы. В 1-ю группу вошли 65 пациенток, уже более 5 лет принимающих терапию эстрогеном и прогестероном (группа МГТ). Вторая группа состояла из 65 женщин, не использующих МГТ. Измерялись уровни липидов в плазме, C-реактивного белка, общий антиоксидантный статус, уровни глюкозы натощак и эстрадиола. Средняя продолжительность применения МГТ составляла 8,4±2,3 года. ДТЛ оказалась больше у участниц из 1-й группы, длительно использующих МГТ (р<0,01) [34].

Основываясь на вышеупомянутых результатах, на сегодняшний день мы можем полагать, что применение МГТ в течение 5 лет и более способно замедлить укорочение ДТЛ в постменопаузе, и в таком случае эффект сопоставим с длительным репродуктивным периодом. Кроме того, не следует пренебрегать рекомендациями по изменению образа жизни и повышению физической активности пациенток. Вероятно, комплексный подход способен не только значительно изменить качество жизни, но и увеличить ее продолжительность. В то же время требуется проведение более масштабных и высококачественных исследований.

Согласно данным различных исследований, эстроген обладает плейотропными свойствами: противовоспалительными, антиоксидантными и, как было упомянуто выше, способствует экспрессии теломеразы. Эстроген через прямой геномный сигнальный путь связывается со своим ядерным рецептором (ERα или ERβ). Комплекс лиганда с рецептором затем перемещается в ядро, и в области эстроген-эффекторных элементов взаимодействует с промотором гена ТОТ.

Что касается противовоспалительной функции эстрогена, то тут не все так однозначно. До сих пор остается неразрешенным парадокс в отношении иммуномодулирующей роли эстрогенов. С одной стороны, эстрогены ингибируют резорбцию костной ткани и подавляют воспаление при ряде хронических воспалительных заболеваний у животных. С другой — моделируют ответ иммунной системы при травмах, сепсисе, а также оказывают провоспалительный эффект при некоторых хронических аутоиммунных заболеваниях у людей [35]. Что касается антиоксидантной функции эстрогенов, то она проявляется, например, в защите нервных клеток от свободных радикалов и эксайтотоксинов- нейромедиаторов, гиперактивирующих NMDA- и AMPA-рецепторы. Так, за счет активации сигнального пути цАМФ и митоген-активируемой протеинкиназы (MAP-киназы) уменьшается влияние на кальциевые каналы и снижается поступление ионов кальция в клетку, таким образом, при воздействии эксайтотоксинов нервная клетка в меньшей степени подвергается апоптозу [36, 37]. Данный эффект также может быть объяснен активацией экспрессии ТОТ. Изобретение активатора теломеразы первоначально выглядело очень многообещающе, однако, как оказалось на практике, его применение не привело к увеличению ДТЛ [38].

Несмотря на то что прямые механизмы воздействия МГТ на ДТЛ остаются под вопросом, косвенно ее применение оказывает положительное влияние. Так, МГТ снижает риск развития ряда хронических заболеваний, что, в свою очередь, частично восстанавливает функции митохондрий, уменьшает уровень системного воспаления и, соответственно, приводит к снижению количества АФК. Следовательно, процесс укорочения ДТЛ замедляется. Частично данный эффект может быть объяснен взаимодействием эстрадиола с рецепторами, расположенными в цитозольной мембране (GPER), что приводит к активации различных каскадов передачи и в том числе — к активации эндотелиальной синтазы оксида азота (eNOS) [39]. Как известно, нарушенная продукция оксида азота вовлечена в патогенез ряда заболеваний: гипертонической болезни, сахарного диабета 2 типа (СД2), ожирения и др.

Считается, что до наступления менопаузы женщины заболевают СД2 реже, чем мужчины. Пик заболеваемости приходится на период постменопаузы. Кроме того, ранняя менопауза ассоциирована с большим риском развития СД2 [40]. Двойное слепое плацебо-контролируемое исследование показало, что в группе пациентов, принимающих МГТ, риск развития СД2 типа составил 6,2%, а в группе плацебо — 9,5% (ОР 0,65; 95% ДИ 0,48–0,89; p=0,006) [41]. МГТ повышает чувствительность рецепторов к инсулину, а также улучшает его секрецию β-клетками поджелудочной железы. Тем не менее МГТ не одобрена FDA для применения с целью профилактики развития СД2, без прямых показаний для ее назначения.

В период менопаузального перехода и в раннюю постменопаузу у женщин наблюдается перераспределение жировой ткани, что приводит к увеличению доли висцерального жира и коррелирует с развитием гипертонии, дислипидемии, инсулинорезистентности и другими метаболическими факторами риска сердечно-сосудистых заболеваний. Наличие избыточной массы тела и ожирения в ряде случаев приводит к более тяжелому течению приливов [42]. Поэтому нередко такие пациентки становятся кандидатами для назначения МГТ. В исследовании The OsteoLaus было показано, что применение МГТ статистически значимо (p=0,03) уменьшает массу висцеральной жировой ткани (ВЖТ), приводит к снижению ИМТ на 1,3 кг и при дальнейшем применении предотвращает набор ВЖТ. В среднем ИМТ, с поправкой на возраст, снижается на 0,9 кг/м2 [43]. Большое исследование по профилактике остеопороза также показало, что прибавка массы тела у женщин в постменопаузе, получавших МГТ, была меньше, чем у женщин, получавших плацебо [44]. Тем не менее проспективное нерандомизированное когортное исследование с участием 671 женщины, за которыми наблюдали в течение 15 лет, показало, что терапия эстрогенами не влияла на массу тела [45]. Отмечается положительное влияние МГТ на соотношение липопротеинов высокой и низкой плотности. Данные эффекты, возможно, связаны с метилированием ДНК под воздействием МГТ [30] и не зависят от возраста пациенток [46]. Однако связь дислипидемии и ДТЛ также неоднозначна. Считается, что слабым протективным эффектом в отношении ДТЛ обладает высокий уровень липопротеинов высокой плотности, а вот уровень липопротеинов низкой плотности, как ни странно, прямого влияния не оказывает [47].

По данным некоторых исследований, МГТ помогает справиться с депрессией и лабильностью настроения в период перименопаузы, особенно пациенткам с сопутствующими вазомоторными симптомами [48][49]. Риск развития депрессии впервые в жизни в этот период составляет примерно 30%; для женщин с депрессией в анамнезе риск увеличивается до 60% [50][51]. В метаанализе, проведенном на основании результатов 38 исследований и 34 347 участников, было показано, что депрессия является значимым фактором риска ускоренного сокращения ДТЛ (величина эффекта Коэна -0,205) [52]. Кроме того, депрессия повышает риск развития ожирения, СД2, сердечно-сосудистых заболеваний, что косвенно также приводит к ускоренному клеточному старению [53]. Таким образом, прием МГТ пациентками с депрессивными расстройствами оказывает множественный положительный эффект на ДТЛ. Тем не менее, до сих пор неясно, является ли психологический стресс причиной или следствием укорочения ДТЛ [54].

## ЗАКЛЮЧЕНИЕ

Начиная с перименопаузального периода в организме женщины возникают и манифестируют многие хронические заболевания, которые оказывают влияние как на качество, так и на продолжительность жизни: увеличение количества гинекологических заболеваний, сердечно-сосудистые заболевания, остеопороз и остеоартроз, ожирение, СД2, депрессия и др. Благодаря доступной профилактике заболеваний, сопровождающих данный этап, на сегодняшний день возможно не только улучшить качество жизни, но и увеличить ее продолжительность.

Понимание молекулярных механизмов, опосредующих протективное влияние МГТ на гомеостаз и целостность теломер, будет полезным для разработки стратегий вмешательства, защищающих теломеры от окислительного стресса и способствующих здоровому старению.

## ДОПОЛНИТЕЛЬНАЯ ИНФОРМАЦИЯ

Источники финансирования. Исследование выполнено в рамках государственного задания «Влияние эпигенетических факторов на течение менопаузы у женщин с эндокринопатиями аутоиммунного генеза в рамках формирования модели “здорового старения”» 2021–2023 г.

Конфликт интересов. Авторы декларируют отсутствие явных и потенциальных конфликтов интересов, связанных с содержанием настоящей статьи.

Участие авторов Мокрышева Н.Г., Андреева Е.Н., Григорян О.Р., Фролова Т.М., Михеев Р.К. — концепция и дизайн исследования; Фролова Т.М., Михеев Р.К., Шереметьева Е.В., Абсатарова Ю.C., Ужегова Ж.А. — сбор и обработка материала; Григорян О.Р., Фролова Т.М., Михеев Р.К., Шереметьева Е.В., Абсатарова Ю.C., Ужегова Ж.А. — написание текста; Мокрышева Н.Г., Андреева Е.Н., Григорян О.Р. — редактирование. Все авторы одобрили финальную версию статьи перед публикацией, выразили согласие нести ответственность за все аспекты работы, подразумевающую надлежащее изучение и решение вопросов, связанных с точностью или добросовестностью любой части работы.
